# Navigating the transition: assessing continuity and loss in endocrinology care

**DOI:** 10.1530/EC-24-0541

**Published:** 2025-03-08

**Authors:** Hatice Nursoy, Erhan Hocaoglu, Muge Yasar, Filiz Mercan Saridas, Kadircan Karatoprak, Ozen Oz Gul, Soner Cander, Canan Ersoy, Elif Ezirmik, Yasemin Denkboy Ongen, Omer Tarim, Erdinc Erturk, Erdal Eren

**Affiliations:** ^1^ Bursa Uludag University Department of Pediatric Endocrinology, Bursa, Turkey; ^2^ Bursa Uludag University Department of Endocrinology, Bursa, Turkey; ^3^ Karasu District Health Directorate, Karasu, Sakarya, Turkey

**Keywords:** adult health care, congenital adrenal hyperplasia, diabetes mellitus, hyperthyroidism, pediatric endocrinology, pediatric healthcare, pituitary disorders, transition, transition from pediatric to adult care

## Abstract

**Background:**

Transition describes preparing children with chronic illnesses for adult healthcare and gradually transferring their care, starting late adolescence. Joint meetings and visits are recommended during this process. This study examines an 8-year transition experience in our clinic, focusing on the differences between adult and pediatric endocrinology practices and the rates of loss to follow-up in specific disease groups.

**Methods:**

Three hundred thirty-five patients evaluated in transition meetings were included. The frequency of visits 1 year before transition, during the first and second years after transition, the number of patients lost to follow-up and disease groups were analyzed.

**Results:**

Among the patients discussed in the transition meetings, 56.3% participated in joint visits. Post-transition, 82.5% of patients continued their follow-up care, while 17.5% were lost to follow-up. There was a statistically significant difference in the number of visits before and after the first year of transition (*P* = 0.000).

**Conclusion:**

To increase adaptation during the transition, informing patients and families about the process at every visit after late adolescence is beneficial. Moreover, scheduling joint visits on flexible dates and increasing the number of joint visits per patient could substantially enhance patient participation and follow-up rates. We observed that patient follow-up frequency was higher in pediatric endocrinology due to differences in pediatric and internal medicine practices.

## Introduction

Transition is a planned and structured process for patients with chronic conditions moving from pediatric to adult healthcare. It focuses on shifting care from a child-centered to an adult-centered approach. This process typically occurs as patients’ progress from adolescence into young adulthood. It is a gradual process requiring the preparation of the patient, family and pediatrician to ensure a successful transition (1). The preparation phase and process are defined as ‘transition’, while the act of handing over patients is referred to as ‘transfer’ (2). Patients with chronic conditions develop a bond with their physicians and healthcare teams during the pediatric period. In pediatric care, the responsibility lies more with the physician and family. This situation will change in adulthood and young adults will be expected to take responsibility for themselves (3). Preparing the patient for adult healthcare (AHC) will reduce the frequency of being lost to follow-up or developing complications related to the relevant health condition during the process (4). Structured approaches have been developed to ensure a successful transition. It is recommended that the transition process be started at the beginning of adolescence (age 12) and that six essential elements be fulfilled. These elements are stated as policy/guide (age 12–14), tracking and monitoring (age 14–18), readiness (age 14–18), planning (age 14–18), transfer of care (age 18–21) and transition completion (age 18–23) (5). The six core elements approach is widely used in the United States. In the United Kingdom, the ‘Ready Steady Go’ program, developed by the National Health Service and adaptable for use across all subspecialties, is implemented. According to this program, the transition process begins at 11–12 years of age and is assessed at each stage (Ready Steady Go) using questionnaires. In the final stage, ‘Hello’, the young person is transferred to the adult care team (6). It is recommended that the adult and pediatric teams hold joint meetings and visits during the transition period (7). In these joint meetings, the clinical summary of the patients is shared with the adult team. It also allows the two teams to discuss treatment recommendations. There are many chronic diseases in pediatric endocrinology practice. Individuals with these chronic diseases should be transferred to adult endocrine clinics after adolescence. Since possible disruptions in follow-up may increase disease-related complications, the transition period should be planned carefully (8, 9, 10).

This 8-year retrospective study aims to examine the transition experience of pediatric and adult endocrine clinics at Bursa Uludag University Hospital, focusing on differences in clinical practices and loss to follow-up rates among disease groups.

## Method

Our university hospital is located in Bursa, one of the largest cities in Turkey. It serves patients from both Bursa and the surrounding provinces, with adult and pediatric services housed within the same building.

### Transition pathway

In Turkey, adult outpatient clinics are only accessible to patients aged 18 and older, and as such, the policy for transition meetings is structured with the guideline ‘Patients who turn 18 will be admitted’.

Our transition process is primarily facilitated through transition meetings and joint visits. Transition meetings are sessions in which the pediatric and adult endocrinology teams come together to discuss and plan the care of patients transitioning from pediatric to adult healthcare. The patient selection criteria for transition meetings were determined as age, clinical stability, the completion of the diagnostic process and patients who regularly attended outpatient clinic visits in the year before the transition period. Families and patients started being informed about the transition and transfer process during pediatric endocrinology visits from the age of 17 onward. The transition decision was made jointly by the pediatric endocrinology physician team, the patient and the family. Transition meetings were held in person on the last Thursday of each month and virtually during the COVID-19 pandemic. The meetings were attended by physicians from both the pediatric and adult endocrinology teams. Before each meeting, the pediatric team discussed the patients scheduled for transition. The patients’ names were emailed to all members of both teams before the meeting. The pediatric team prepared and presented detailed clinical summaries, which were reviewed by the adult team beforehand. The patients’ clinical histories, diagnoses and treatments were thoroughly examined during the meetings. Both teams noted treatment recommendations. After the meeting, patients were called and informed about the joint visit date. On the first Monday of the month following the week of the transition meeting, patients were seen together by the adult and pediatric endocrinology teams at the adult endocrinology outpatient clinic and this meeting was referred to as a joint visit. After this stage, the follow-up of the patients was transferred to adult endocrinology. Patients who did not attend the joint clinic were informed about the transition during their subsequent initial pediatric visits and were directed to the adult endocrinology clinic for follow-up care. As these patients had already been presented to the adult endocrinology team in advance, no additional meetings were held. After the transition, patients who applied to pediatric endocrinology were classified as ‘reapplied’.

### Patient selection and analysis

Three hundred thirty-five patients evaluated in the transition meetings between January 2016 and December 2023 were included in the study. No patients were excluded based on ethnic background. However, despite the provision of comprehensive and free healthcare services to refugees in Turkey, access to university hospitals is restricted through a referral system, which considerably limits applications from refugees or non-citizens. The patient population is relatively homogenous, with similar characteristics in terms of middle to upper socioeconomic and sociocultural levels. Healthcare insurance for all patients is covered by the government until the age of 18.

Since congenital and acquired hypothyroidism were followed up in internal medicine instead of endocrine outpatient clinics in our center, these patients were not evaluated in transition meetings and joint visits. They were directed to internal medicine outpatient clinics when the transition time came. The visit frequency, number of patients lost to follow-up and disease groups were analyzed for the year before, the first and the second year after the transition. Patients who were called for transition but did not have any adult endocrinology appointments for the 2 years after the transition were considered lost to follow-up.

### Ethics

Patient files were retrospectively scanned into a digital environment. Ethics approval was obtained from the Local Ethics Committee (2023-23/34).

### Statistical analysis

Descriptive statistics for continuous data were presented with mean, standard deviation, median and minimum–maximum values and categorical data with numbers and percentages. The normal distribution of continuous data was assessed with Kolmogorov–Smirnov and Shapiro–Wilk tests. For the comparison of two dependent groups with nonparametric characteristics, the Wilcoxon test was employed, while the Friedman test was used for comparisons involving more than two dependent groups. Post-hoc analyses were performed to identify the source of significant differences among the groups. Statistical significance was considered at *P* < 0.05 within a 95% confidence interval. The IBM SPSS version 21.0 was used for statistical analyses.

## Results

Of the 335 patients, 53.1% (*n* = 178) were female. The diabetes group was the largest, comprising 39.9% (*n* = 134) of the sample. In the diabetes group, 121 patients had type 1 diabetes mellitus (DM), three had type 2 DM and ten had other diabetes diagnoses, such as cystic fibrosis-related DM and maturity-onset diabetes of the young (MODY). Sixty patients were followed with pituitary disorders and 31 patients with adrenal diseases (23 congenital adrenal hyperplasia (CAH) and eight non-CAH primary adrenal insufficiency) (Table 1).

**Table 1:** Demographic characteristics, follow-up status by disease groups and outpatient clinic appointments.

		Percentage (%)	*n* *	Follow-up	
				Lost follow-up	Continue follow-up
Sex	Female	53.1	178		
	Male	46.9	157		
Follow-up	Lost follow-up	17.5 (female 60.4%)	48		
	On follow-up	82.5	227		
Diseases	Diabetes (type 1 DM, type 2 DM, cystic fibrosis-related DM, etc.)	39.9	134	20.0	80.0
	Pituitary disorders (hypopituitarism, prolactinoma, surgery-related hypopituitarism, pituitary adenoma, diabetes insipidus, etc.)	17.9	60	20.7	79.3
	Adrenal diseases (congenital adrenal hyperplasia, Addison disease, etc.)	9.2	31	4.3	95.7
	Hypogonadism	6.0	20	5.6	94.4
	Papillary thyroid carcinoma	5.1	17	0.0	100.0
	Turner syndrome	4.8	16	7.7	92.3
	Hyperthyroidism	3.3	11	30.0	70.0
	Osteogenesis imperfecta	1.2	4	0.0	100.0
	Hypoparathyroidism	1.2	4	0.0	100.0
	Others^†^	11.6	39	30.0	70.0
Number of patients who applied to joint visits	No	43.8	147		
	Yes	56.3	189		
Patients who reapplied to pediatric endocrinology outpatient clinics after transition	No	76.1	252		
	Yes	23.9	79		

* Patients who completed the 2-year follow-up period were included in the analysis.

^†^ Disorders of sex development, hypophosphatemic rickets, McCune Albright syndrome, etc.

Of the patients who were discussed and evaluated during the joint meetings and invited to joint visits, 56.3% attended the joint visits. Following the transition, 23.9% of these patients continued to visit the pediatric endocrinology clinics. Patients who returned to the pediatric clinics had a 22.7% participation rate in joint visits. Of the patients who returned to pediatric endocrinology, 31.6% had a diagnosis of diabetes and 18.9% had pituitary disorders. The distribution of diseases was similar to the overall diagnostic distribution of all patients. 82.5% of the cases continued to be followed up, while 17.5% were lost to follow-up. Among patients who completed 2 years of follow-up after the transition, 13.1% attended follow-up in the first year but discontinued follow-up thereafter.

The mean age of patients at the time of transition was found to be 18.36 ± 0.63 years. The mean number of appointments in the year before the transition was 3.79 ± 1.58. The mean number of appointments in the year after the transition was 3.40 ± 1.65 in the first year and 2.94 ± 1.63 in the second year (Tables 2 and 3). A statistically significant difference was found between the number of visits of the patients in the year before the transition and the first year after the transition (Table 2). The median number of visits was higher in the year before the transition (*P* = 0.000) (Table 2). The number of follow-ups in the first year after transition was significantly higher than that in the second year (*P* = 0.000) (Tables 3 and 4).

**Table 2:** Comparison of follow-up visit numbers in the year before the transition and the first year after the transition.

	*n* ^†^	Mean	SD	Median	Minimum	Maximum	*P*
Number of visits in the year before the transition	237	3.79	1.58	4.00	1.00	8.00	0.000*
Number of follow-ups in year 1	237	3.40	1.65	3.00	1.00	10.00	

* Wilcoxon test was used.

^†^ Patients who attended follow-up visits both in the year before the transition and in the first year post-transition were included in the analysis.

**Table 3:** Comparison of visit numbers in the first and second years post-transition.

	*n* ^†^	Mean	SD	Median	Minimum	Maximum	*P*
Number of follow-ups in year 1	159	3.60	1.78	3.00	1.00	10.00	0.000*
Number of follow-ups in year 2	159	2.94	1.63	3.00	1.00	9.00	

* Wilcoxon test was used.

^†^ Patients who attended follow-up visits in both the first and second years post-transition were included in the analysis.

**Table 4:** Pairwise comparison of the year before transition, the first year post-transition and the second year post-transition.

	*n* ^†^	Mean	SD	Median	Min	Max	*P*	Pairwise
Number of visits in the year before the transition	157	4.01	1.56	4.00	1.00	8.00	0.000*	Year before transition–first year post-transition (*P* = 0.028)
Number of follow-ups in year 1	157	3.55	1.73	3.00	1.00	10.00		First year–second year (*P* = 0.002)
Number of follow-ups in year 2	157	2.92	1.62	3.00	1.00	9.00		Year before transition–second year post-transition (*P* = 0.000)

* Friedman test was used.

^†^ Patients who attended follow-up visits in the year before the transition and in the first and second years post-transition were included in the analysis.

Among the patients with DM and pituitary diseases, 20% were lost to follow-up. In the hyperthyroid patients, 30% were lost to follow-up. None of the patients with papillary thyroid cancer (PTC), osteogenesis imperfecta (OI) and hypoparathyroidism were lost to follow-up. An analysis of all disease groups showed that patients with hyperthyroidism, type 1 DM and pituitary disorders had the highest risk of discontinuing follow-up care (Table 1).

## Discussion

Our study found a 43.8% non-attendance rate at the first joint visit, with a 17.5% overall loss to follow-up. Prodam and coworkers reported an overall loss to follow-up rate of 35.3%, with most of the dropouts occurring after the first joint visit (11). The low participation rate in the first joint visit in our study is believed to be associated with the lack of scheduling flexibility and the short notification period provided to patients (2 business days). This assumption is further supported by the fact that these patients subsequently continued with their follow-up appointments. In addition, relocating to a different city for university may also influence the rates of loss to follow-up. Early and consistent patient and family education on transition, starting in late adolescence, can reduce follow-up loss. Flexible scheduling for joint visits may increase attendance.

In our study, 23.9% of patients returned to pediatric endocrinology clinics following the transition. There are patient, family and pediatrician perspectives on difficulty in the transition process. In a study examining the transition from the perspective of an internist, it was observed that pediatricians had difficulty letting go of the patient (12). In addition, it was noted that patients and families were afraid and reluctant to leave the pediatrician and pediatric healthcare team with whom they were accustomed and had established a long-term bond (13, 14). Based on the data from this study, particularly the frequency of reconsultations by patients at pediatric endocrinology clinics, it is suggested that initiating joint visits with the adult endocrinology team for patients aged 16–17, while still under pediatric endocrinology care, could be beneficial, as early contact with the AHC team before transition can enhance the process (13, 15).

The higher follow-up rate in pediatric endocrinology compared to adult endocrinology might be attributed to differences in practice approaches. The fact that treatment simplification and longer visit intervals were recommended to increase compliance after the transition is consistent with this situation (16). Diabetes follow-up rates during transition were reported as 75% for type 1 diabetes in the literature (17). In our study, considering all diabetes cases (type 1 diabetes, type 2 diabetes, cystic fibrosis-related diabetes, etc.), we found an 80% follow-up rate, indicating success. However, evaluating each diabetes type individually and comparing HbA1c levels could provide more insights.

Our study considered pituitary disorders as a heterogeneous group (panhypopituitarism, prolactinoma, surgery-related hypopituitarism, pituitary adenoma, diabetes insipidus, etc.). Pituitary disease follow-up loss was 20%. Due to the long-term nature of these conditions, minimizing follow-up loss is crucial. Reviewing retesting needs before the transition in pituitary hormone deficiencies is necessary (18).

CAH follow-up loss was reported at 50% during the transition (19). Our study found a 4% loss for CAH and non-CAH primary adrenal insufficiency, likely due to the region’s limited tertiary healthcare institutions until recent years. Future studies should reexamine these rates as healthcare availability changes.

There is limited literature on transition in thyroid diseases. Adolescence and transition periods may cause delays in follow-up. In this case, it may negatively affect fertility, especially in women with hypohyperthyroidism (20). In our study, 30% of the ten hyperthyroid patients were lost to follow-up. This rate is higher than that of other endocrine diseases in the study. There is no literature on follow-up status for hyperthyroidism and more studies are needed. In our study, none of the patients with PTC (*n* = 13), OI (*n* = 3) or hypoparathyroidism (*n* = 2) were lost to follow-up during the transition period. Although the number of patients with OI and hypoparathyroidism is too small to comment on, it is worth emphasizing that all of the patients with PTC remained on follow-up. Childhood cancer survivors should be followed up for long-term complications and recurrence (21). The literature on this subject has focused on leukemia, lymphoma and brain tumors, which are most common in childhood (22). Unlike other childhood cancers, pediatric endocrinologists follow PTC. The endocrinologist leads the multidisciplinary team. Since long-term follow-up is required for recurrences and secondary cancers due to radioactive iodine, persistent hypoparathyroidism and hypothyroidism, it is essential to make the transition appropriate (23, 24). The fact that PTC patients are not lost to follow-up may be due to families and patients taking follow-up more seriously, possibly due to the cancer diagnosis. Further studies are needed on long-term follow-up of patients with PTC after transition.

Limitations of our study: first, in our center, the transition process begins after the age of 18, and joint visits are conducted on a fixed day. These situations appear to negatively affect adherence to the transition process and participation rates in joint visits. Second, it is a retrospective study, which may introduce bias in data quality, collection and selection. Third, as we focused exclusively on patients who underwent a structured transition process, we needed more data on those who were transferred to adult care without such a process. Finally, we did not analyze patients who initially continued their follow-up after the transition but were later lost to follow-up.

Strengths of our study: this study is one of the few studies examining the frequency of follow-ups of transition patients in endocrinology and the rates of loss to follow-ups according to diseases. Moreover, this study is particularly significant as there is a limited body of research on this topic from Turkey.

## Conclusions

It is crucial for individuals with chronic diseases who need to transition to AHC to be both physically and mentally prepared. This preparation should also include the family and healthcare teams. Commencing joint visits at the ages of 16 or 17, providing patients with flexible scheduling options and ensuring that patients are informed early about the dates of joint visits could improve patient participation and decrease the risk of discontinuation from follow-up care. Detailed analyses of the reasons for patients returning to pediatric care are required along with the development of strategies to address this issue. Further research is needed in endocrinology to examine the variations in loss to follow-up rates among different disease groups and to explore the underlying reasons.

## Declaration of interest

The authors declare that there is no conflict of interest that could be perceived as prejudicing the impartiality of the work.

## Funding

This work did not receive any specific grant from any funding agency in the public, commercial or not-for-profit sector.

## Data availability

The data that support the findings of this study are not openly available due to institutional policy, but will be available from the corresponding author upon reasonable request.

## Ethics approval consent to participate

The Ethical Board at Bursa Uludag University approved the study with protocol no. 2011-KAEK-26/808 and approval no. 2023-23/34, with a waiver of the study subjects’ consent as per the institutional policy for retrospective studies.
